# Unusual causes of perineal trauma in paediatric patients: lessons learned from a high-volume colorectal clinic in a low-and-middle-income country

**DOI:** 10.1007/s00383-025-06075-x

**Published:** 2025-06-18

**Authors:** Francesca Palmisani, Keitumetse Teko, Catterina Bebington, Giulia Brisighelli

**Affiliations:** 1 Department of Paediatric Surgery, Chris Hani Baragwanath Academic Hospital, Faculty of Health Sciences, University of the Witwatersrand, Chris Hani Road, Diepkloof, Johannesburg, South Africa; 2 Department of Paediatric Surgery–Medical University of Vienna, Währinger Gürtel 18-20, 1090 Vienna, Austria; 3 Johannesburg Paediatric Colorectal Clinic (JPCC), Department of Paediatric Surgery, Chris Hani Baragwanath Academic Hospital, Faculty of Health Sciences, University of Witwatersrand, Chris Hani Road, Diepkloof, Johannesburg, South Africa

**Keywords:** Perineal trauma, Low-middle income country, Acquired colorectal conditions, Paediatric colorectal surgery

## Abstract

**Purpose:**

In low-and-middle-income countries (LMIC), a non-negligible number of cases of paediatric perineal trauma is observed. Trauma can arise from less conventional mechanisms such as traditional enemas and flame burns, alongside blunt and penetrating injuries. The data on surgical management and long-term outcomes are limited. This study aims to evaluate surgical treatment and continence outcomes post-reconstruction.

**Methods:**

A retrospective review was conducted at Johannesburg’s Chris Hani Baragwanath Academic Hospital (2018–2025). Cases of sexual abuse were excluded. Patients were stratified based on whether they required surgical reconstruction. Data analysed included demographics, injury cause, procedures, and long-term continence via the Krickenbeck questionnaire.

**Results:**

Of 20 patients, 10 underwent reconstruction. In the conservative group, 7 (70%) needed a colostomy for sepsis-free recovery. The median age was 3.5 years in the reconstruction group versus 6.6 years in the conservative group (*p* = 0.039). Traditional enemas caused 60% of injuries in the reconstruction group, while blunt and penetrating trauma was predominant (70%) in the conservative group (p = 0.05). Reconstruction included Swenson pull-through (7), PSARP (2), and perineal body reconstruction (2). The median follow-up was 4 years: 75% of patients achieved continence.

**Conclusion:**

Established anorectal reconstruction techniques are effective for traumatic perineal injuries, providing good cosmetic and continence outcomes.

## Introduction

In low- and middle-income countries (LMICs), the overall incidence of trauma in children is higher than in high-income settings, largely due to factors such as poverty, lack of safety infrastructures and limited injury prevention measures. Among these injuries, perineal trauma accounts for 0.2% to 8% of cases and can present in a variety of forms. Nonaccidental injuries, particularly sexual abuse, are responsible for up to one third of perineal trauma cases; however, this figure is likely underestimated due to limited access to care and the frequent underreporting of abuse.

Although most literature focuses on sexual abuse and accidental trauma, such as motor-vehicle accidents and falls from height, a small but important subset of cases arises from less conventional or unusual mechanisms. These include injuries from traditional, hot water and contrast enemas, as well as thermal injuries, such as flame or hot water burns. Although rare, these mechanisms pose significant diagnostic and management challenges and are sparsely described in the paediatric literature [1, 2].

The use of traditional and hot-water enemas dates back into African history, with the widespread use of herbal medication from traditional healers, and, more recently, homemade mixtures given for therapeutic purposes or cultural rituals [3, 4]. Although uncommon, the administration of hot-water enemas as disciplinary measure or potty-training technique for children still occurs in isolated cases, and the resulting injuries should be regarded as non-accidental. Documented complications, in both children and adults, include profuse diarrhoea, infections, dehydration, proctocolitis, rectal strictures, rectal wall injury, rectal perforations, and in severe cases organ dysfunction, with one case of death published [1, 4, 5]. However, their complications and surgical management have mainly been described in the adult population, with very little data on the surgical approach to the paediatric population [1].

Flame and hot-water burns to the perineum have been previously described in the literature, as well as the use of temporary diverting colostomies for their management [2]. However, to our knowledge, no acquired imperforate anus secondary to the burns has ever been reported before.

Evidence regarding the long-term outcomes of paediatric perineal trauma, especially in cases of extensive anorectal injury requiring surgical intervention, is limited [6]. The few available studies on children report an incidence of up to 19–50% of faecal incontinence and up to 11% of anal stenosis, depending on the extent of injury [6, 7].

The aim of this study was to retrospectively review the surgical management and outcome of paediatric patients that presented to our centre with complications arising from perineal trauma, including unusual causes.

## Materials and methods

We retrospectively reviewed all electronic and hard records of patients who presented with perineal trauma to the Johannesburg Paediatric Colorectal Clinic (JPCC) of Chris Hani Baragwanath Academic Hospital (Johannesburg, South Africa) in the period between January 2018 and March 2025. All cases of confirmed or suspected sexual abuse were excluded from the analysis, as these had been previously reported by our group [4]. The data regarding demographics, age, mechanisms and type of injury, surgical management and postoperative course were analysed.

The patients were divided into two groups, according to weather or not they required surgical reconstruction. Statistical analysis to evaluate differences between the two groups was performed using a *t* Student test. *P* value of < 0.05 was considered statistically significant.

Functional bowel outcomes were assessed in all patients who underwent surgical reconstruction and were older than three years of age at the time of follow-up. The Krickenbeck questionnaire was used to assess the presence of voluntary bowel movements, soiling or constipation requiring any form of bowel management postoperatively.

Ethical approval was granted by the Ethics Committee of the University of Witwatersrand, under the protocol M190508.

## Results

In the study period, 32 patients presented to JPCC with injuries resulting from perineal trauma. Sexual abuse cases accounted for 12 cases (37%). As their surgical management had been already previously published elsewhere by our group [4], these cases were excluded from the current analysis. The remaining 20 patients were included in the study.

The cohort showed an equal distribution in gender (male-to-female-ratio 1:1) with a mean age at presentation of 4.7 years (range 0–11).

Detailed clinical and demographic data are shown in Table 1.

**Table 1:** Mechanism and extent of perineal injury in 20 patients with associated surgical strategies

Injury location	Type of injury	Mechanism of injury	Surgical Strategy	Number of patients	Age at presentation (years)
Genitourinary system	Vaginal avulsion	Traditional enema (delivered in the vagina)	Conservative treatment	1	–
	Rectovaginal tear	Straddle injury from blunt trauma	Colostomy + conservative treatment	1	5
Perineum and anal canal	Necrotizing soft tissue infection	Traditional enema	PSARP + Swenson Pull-through	1	2
	Full thickness burns with sphincter disruption	Flame burns	PSARP	1	3
	Perineal tear	Straddle injury from fall	Perineal body repair	2	8
			Colostomy + conservative treatment	1	8
	Perineal tear	Straddle injury unknown cause	Conservative treatment	1	–
			Colostomy + conservative treatment	1	5
	Perianal laceration	Straddle injury from bicycle accident	Colostomy + conservative treatment	1	6
	Anal avulsion	Blunt trauma (PVA)	Colostomy treatment	1	9
	Penetrating perineal injury	Bicycle spoke- non accidental	Colostomy + conservative treatment	1	11
Ano-rectum	Full-thickness burn 5 cm from the anal verge	Traditional enema	Swenson Pull-through	1	1
Rectum	Rectal necrosis	Traditional enema	Swenson Pull-through	2	6 2
	Extraperitoneal rectal perforation	Contrast enema (iatrogenic)	Swenson Pull-through	1	0
	Rectal perforation	Traditional Enema	Colostomy + conservative management	1	9
Recto-sigmoid	Necrosis of sigmoid and rectum down to anterior anal sphincters	Traditional enema	Swenson Pull-through	1	0
	Focal sigmoid perforation	Traditional enema	Colostomy at the level of the perforation (no reconstruction needed)	1	0
Descending/transverse colon	Full thickness burns up to distal transverse colon	Traditional enema	Swenson pull-through	1	2

*PVA* pedestrian vehicle accident

Surgical reconstruction was required in ten patients (10/20, 50%), due to the complex nature of the injuries to the sphincters and/or bowel. In these cases, the mean age at presentation was 3.5 years (range 0–8 years), with male predominance (6/10, 60%).

Traditional or hot-water enemas were the leading cause of injury in this group (6/10, 60%). Further mechanisms of trauma included straddle injuries secondary to falls (2/10, 20%), perianal flame burns caused by a shack fire (1/10, 10%) and one iatrogenic injury during the delivery of a contrast enema (1/10, 10%).

**Fig. 1:**
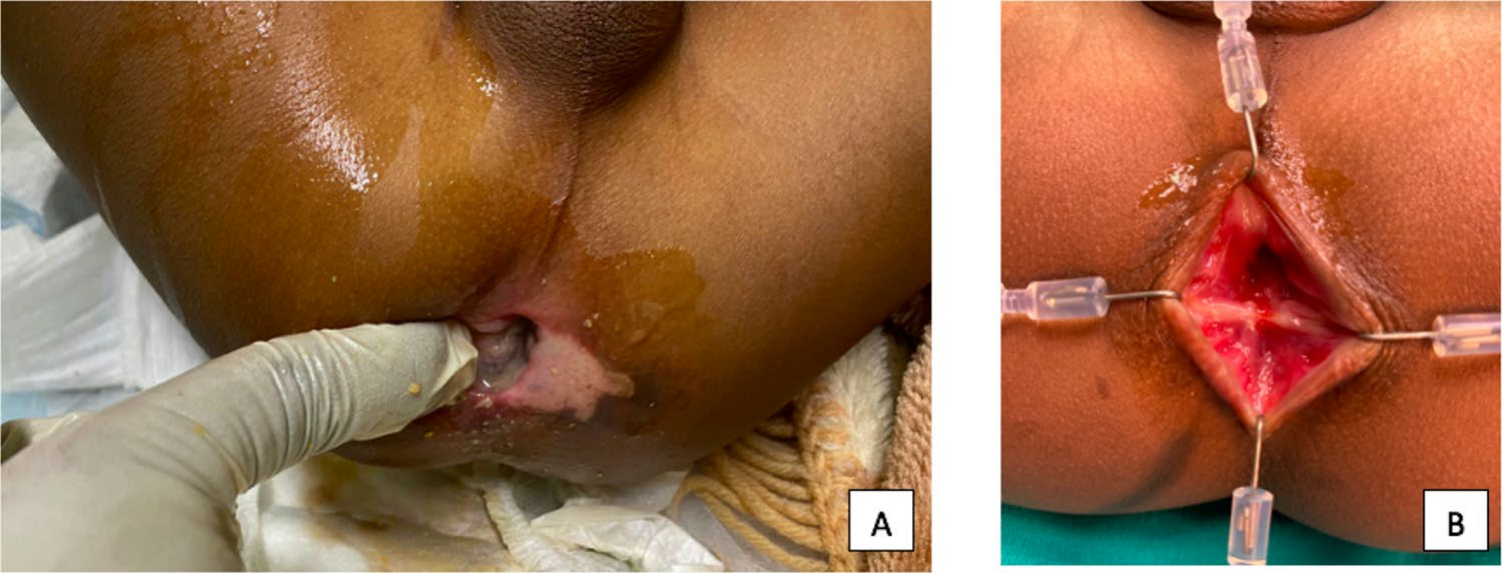
Injuries resulting from a traditional enema. Figure 1a illustrates the injuries preoperatively; Fig. 1b illustrates the postoperative result after Swenson Pull-through

**Fig. 2:**
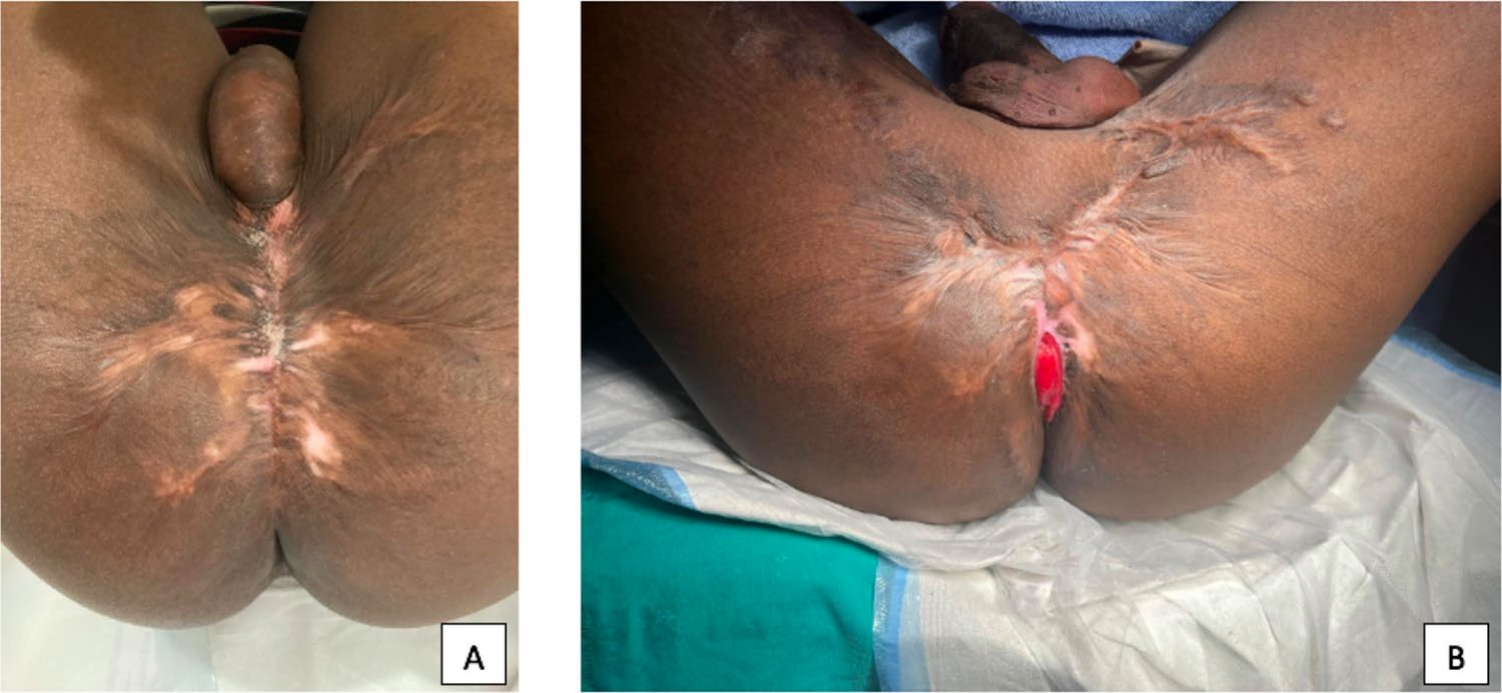
Injuries resulting from a flame burns. Figure 2a illustrates the injuries preoperatively (complete disruption of the sphincters with scarring and complete stricture of the anus); Fig. 2b illustrates the postoperative result after PSARP

**Fig. 3:**
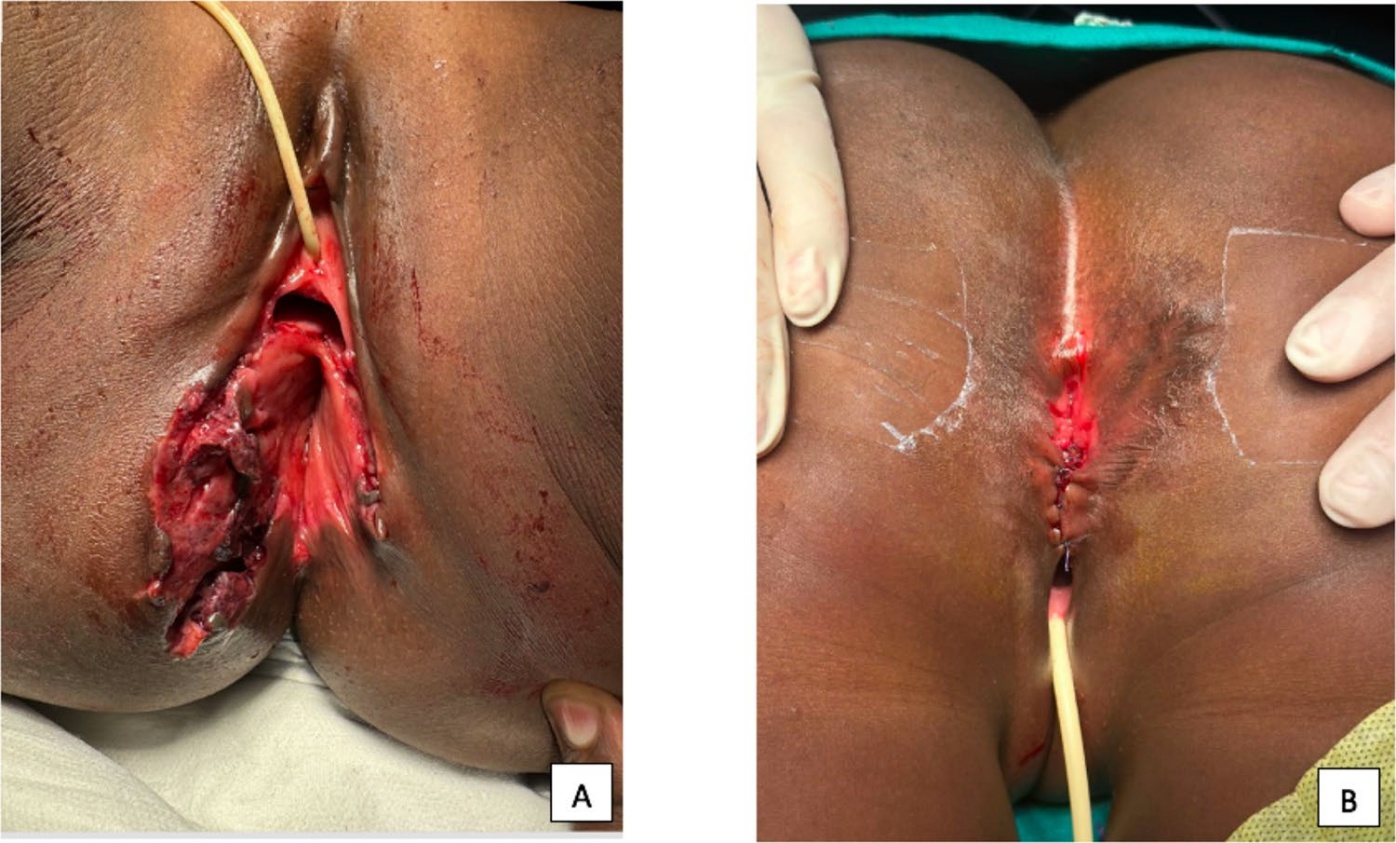
Straddle Injuries resulting from fall. Figure 3a illustrates the injuries preoperatively; Fig. 3b illustrates the postoperative result after perineal body repair

The surgical procedures were performed as follows:

- Swenson pull-through was performed in seven cases (7/10, 70%), where the injury was located to the recto-sigmoid or descending colon – five from traditional or hot-water enemas and one from a contrast enema. One patient also underwent a concurrent PSARP due to acquired imperforated anus (Fig. 1).
- Posterior sagittal anorectoplasty (PSARP) was performed in two cases (2/10, 20%), where the lesion involved the anal canal (one from flame burns and one from a traditional enema). The latter was combined with a pull-through of his colostomy, as previously mentioned (Fig. 2).
- Perineal body repair was performed in the two patients with straddle injuries secondary to blunt trauma (2/10, 20%). This surgical technique has been previously described by our group in the treatment of perineal trauma secondary to sexual assault [8]. Following the principles of a PSARP, it consists in the careful mobilization of the posterior vaginal wall and the anterior rectal wall until the fibroalveolar plane is reached. At this point the sphincter complex is assessed with a muscle stimulator and the perineal body is reapproximated with interrupted sutures (Fig. 3).

The median time from presentation to reconstruction was 7.5 months (range: 0–213 months).

All patients received a covering colostomy that was subsequently closed after successful recovery from the reconstructive surgery.

The remaining 10 cases presented with injuries that healed with conservative treatment. These patients had a mean age at presentation of 6.6 years (0–11), and a female predominance (6/10 60%). A temporary diverting colostomy was required in seven of them (7/10, 70%), to allow for sepsis-free recovery. The main cause of injury in this group of patients was blunt force or penetrating trauma with straddle injuries, which occurred in seven cases (7/10, 70%). Traditional enemas were responsible for the remaining three cases (3/10, 30%). After initial evaluation under anaesthesia (EUA) the decision to perform a covering colostomy was taken according to the degree of injury.

Table 2 summarizes the mechanisms of injury, and the operations performed.

**Table 2:** Summary of the mechanisms of injury and the operations performed

	Only EUA	EUA + Stoma	EUA + Stoma + PSARP	EUA + Stoma + Pullthrough	EUA + Stoma + Pullthrough + PSARP	EUA + Stoma + perineal body repair
Traditional enema	1	2	0	5	1	0
Blunt/penetrating trauma	2	5	0	0	0	2
Flame burns	0	0	1	0	0	0
Contrast enema (iatrogenic)	0	0	0	1	0	0

The two groups were found to have a statistically significant difference in age at presentation (p value: 0.039), and mechanism of injury (enema vs straddle, *p* value 0.05).

Continence outcomes were evaluated postoperatively in the patients that required surgical reconstruction and are currently above the age of three, which is generally considered to be the physiological age for potty training. Of the eight patients in this age group, six (6/8, 75%) were found continent with no need for any bowel management program (Krickenbeck), one is still undergoing toilet training and one was lost to follow up.

The median length of follow up was four years (range: 0–18).

## Discussion

Perineal trauma in children comprises a wide spectrum of injuries that may vary from minor perineal lacerations to extensive injuries involving the perineum, ano-rectum and genitourinary system [1, 9]. The more severe cases represent an exceptionally rare subgroup, for which established guidelines are still lacking.

Although clinical tools, such as the Onen’s classification for genital injuries in children, illustrated in Table 3 [11], have the purpose to categorize the extent of the injuries and guide the appropriate management, the authors believe that their applicability in the context of paediatric trauma is limited. Especially in case of rare and uncommon causes of trauma, such as traditional or hot-water enemas, the extent of the injury typically does not fall into the given categories and is therefore not suited for clinical practice.

**Table 3:** Onen’s classification for genital injury in children

Genital Injury Score (GIS)	Extent of Injury
I	Isolated genital laceration below hymen or limited to penile and/or scrotal skin
II	Isolated genital laceration including hymenor tunica dartos of scrotum and/or Bucks fascia of penis
III	Isolated genital laceration including vagina or testis and/or penile cavernous or distal urethra
IV	GIS II or III injury plus partial tear of anorectum
V	Grade III injury plus complete tear of anorectum

Examination under anaesthesia (EUA) and faecal diversion via a colostomy are traditionally recommended in case of rectal injuries. Primary repair has been however described with excellent results, in selected cases of extraperitoneal injuries [10]. Shorter length of hospitalization as well as a lower complication rates have been described in this group of patients, although this may reflect the less severe nature of injuries in those managed without diversion. Full-thickness intraperitoneal injuries typically require a diverting stoma. Notably, Russel et al. described successful primary repair without diversion also in stable patients with isolated intraperitoneal rectal injury or anal injuries without significant soft tissue loss or sphincter destruction [6].

The specific surgical techniques used in extensive perineal trauma have been sparsely described in literature [6, 12]. A single institution reported on the use posterior sagittal anorectoplasty (PSARP) for three cases of devastating perineal trauma with sphincter disruption [12] and Russel et al. reported on the use of a Swenson endorectal pull through in one case of destructive pelvic and spinal injury due to gunshot trauma [6].

In our experience, the Swenson pull-through was the most common surgical technique employed for the repair (7/10 70%). This is most probably related to the differences in the mechanisms and type of injury in our patient population. Although other institutions report on motor-vehicle accidents and falls from height being the most common causes of extensive injury [6, 7], our centre most commonly dealt with trauma resulting from traditional enemas (6/10, 60%). These practices can indeed be responsible for extensive injuries, which in some cases caused full-thickness necrosis as proximally as the descending colon. Moreover, such injuries were encountered in a much younger population, while literature describes perineal trauma to be most common between the ages of 5 and 9 years [13]. In our experience, this age group was more prone to present with straddle injuries from blunt force or penetrating trauma, which healed conservatively, with or without the need of a diverting stoma. Differently from the previous reports, no primary repair was performed in our cohort. Although our retrospective design and the lack of a standardized grading system limit our ability to assess whether primary repair would have been feasible in selected cases, our clinical decisions were informed by context-specific considerations. Our environment is burdened by an increased risk of sepsis, which necessitates a cautious approach to infection control. In our experience, primary repair of both acquired and congenital anorectal malformations is frequently associated with a high rate of postoperative complications in this context [14].

Long-term faecal continence after perineal trauma has been sparsely investigated so far. Incontinence rates have been reported to be as high as 19–50% [6, 7]. The largest series on the subject comprises 21 patients managed at a single institution [6]. None of the patients had injuries beyond the rectum. All patients with isolated anal injuries were continent after primary repair, whilst of the four patients with extensive full thickness injuries to the ano-rectum or destructive injuries to the sphincters only two are currently continent to stools.

In contrast, our study shows promising outcomes: among the reconstructed patients over age three, 75% were continent without requiring bowel management. We attribute this in part to the adaptation of surgical techniques originally developed for the management of congenital anorectal malformations and Hirschsprung’s disease, suggesting that such approaches may offer favourable outcomes in acquired injuries as well.

## Conclusion

Severe perineal trauma in children is rare and often context-specific, particularly in low- and middle-income countries (LMICs), where unique social determinants contribute to uncommon injury mechanisms. Culturally rooted practices—such as traditional enemas—can result in devastating injuries, underscoring the urgent need for public health education to reduce harmful practices and promote safer, evidence-based alternatives.

Through the adaptation of the existing surgical techniques and the establishment of standardized protocols for injury assessment and management, we can adapt and improve interventions that can be locally relevant and sustainable. A multidisciplinary approach involving surgical innovation, public health policy, community education and awareness is crucial in addressing the complex needs of this vulnerable population.

## Data Availability

No datasets were generated or analysed during the current study.
